# A comparison of phase imaging and quantitative susceptibility mapping in the imaging of multiple sclerosis lesions at ultrahigh field

**DOI:** 10.1007/s10334-016-0560-5

**Published:** 2016-04-25

**Authors:** Matthew John Cronin, Samuel Wharton, Ali Al-Radaideh, Cris Constantinescu, Nikos Evangelou, Richard Bowtell, Penny Anne Gowland

**Affiliations:** Brain Imaging and Analysis Centre, Duke University, Durham, NC 27710 USA; Sir Peter Mansfield Imaging Centre, School of Physics and Astronomy, University of Nottingham, University Park, Nottingham, NG7 2RD UK; Sir Peter Mansfield Imaging Centre, Queens Medical Centre, University of Nottingham, Nottingham, NG7 2RD UK; Department of Medical Imaging, Faculty of Allied Health Sciences, Hashemite University, Zarqa, Jordan

**Keywords:** Magnetic resonance imaging, Multiple sclerosis, White matter, Iron, Myelin

## Abstract

**Objective:**

The aim of this study was to compare the use of high-resolution phase and QSM images acquired at ultra-high field in the investigation of multiple sclerosis (MS) lesions with peripheral rings, and to discuss their usefulness for drawing inferences about underlying tissue composition.

**Materials and methods:**

Thirty-nine Subjects were scanned at 7 T, using 3D *T*_2_*-weighted and *T*_1_-weighted sequences. Phase images were then unwrapped and filtered, and quantitative susceptibility maps were generated using a thresholded k-space division method. Lesions were compared visually and using a 1D profiling algorithm.

**Results:**

Lesions displaying peripheral rings in the phase images were identified in 10 of the 39 subjects. Dipolar projections were apparent in the phase images outside of the extent of several of these lesions; however, QSM images showed peripheral rings without such projections. These projections appeared ring-like in a small number of phase images where no ring was observed in QSM. 1D profiles of six well-isolated example lesions showed that QSM contrast corresponds more closely to the magnitude images than phase contrast.

**Conclusions:**

Phase images contain dipolar projections, which confounds their use in the investigation of tissue composition in MS lesions. Quantitative susceptibility maps correct these projections, providing insight into the composition of MS lesions showing peripheral rings.

## Introduction

*T*_2_*-weighted gradient echo sequences are often used to study multiple sclerosis (MS) due to the high signal to noise ratio (SNR) and good contrast [1–10] that they provide. With the rising availability of high (3 T) and ultra-high field (7 T and higher) MRI systems, the phase data associated with the *T*_2_*-weighted magnitude images are increasingly being used both as an adjunct to conventional magnitude images [10–13], or in combination with them to produce susceptibility-weighted (SWI) images [7, 14, 15], as they provide a complimentary contrast mechanism.

In recent years, phase and SWI images have been used to study the variation in iron levels in different anatomical brain regions, mainly deep brain nuclei, with age and gender [13, 16], as well as changes in iron levels in MS [3, 7, 10]. However, the use of phase contrast as a qualitative or quantitative measure of iron content assumes a direct relationship between signal phase and local iron levels. This assumption is flawed since a change in magnetic susceptibility, such as a local increase in iron concentration, produces a change in the magnetic field (and hence phase) that is not localized to the susceptibility perturbation, but instead is dipolar in nature, causing both positive and negative field/phase perturbations in the surrounding region [14]. This non-local relationship can lead to incorrect inferences being drawn about local iron levels based on phase or SWI images. The non-local nature of the phase contrast can be overcome by quantitative susceptibility mapping (QSM) [17–19], which produces maps of the local variations in the tissue susceptibility that are responsible for the measured phase changes. The QSM technique has, amongst other applications, been used in vivo to measure changes in the magnetic susceptibility of the basal ganglia in MS patients [1, 2]. While this technique offers a local contrast directly linked to the physical property of magnetic susceptibility, it relies on accurate measurement of the local field perturbations originating only within the imaging volume. For this reason, care must still be taken in the choice of filtering algorithms and parameters for processing of the phase data.

The white matter lesions occurring in MS are sometimes surrounded by rings on *T*_2_*-weighted magnitude and phase images. It has been suggested that these rings may be a marker of local changes in iron content [5, 7, 10]. However, this MR signature could also result from a local variation of the myelin density, or even from changes in tissue microstructure [20], and so the origin of peripheral rings remains a matter of some debate. The ability to detect iron changes around lesions would be useful in understanding the pathogenesis of MS lesions and in tracking disease progression. In previous work, phase and SWI images have been used to investigate the prevalence and nature of peripheral rings in MS lesions [3, 7, 10].

The aim of the work described here was to compare the depiction of white matter MS lesions in phase and QSM data, taking advantage of the high resolution (0.5 mm, isotropic) achievable in vivo at 7 T. The prevalence of peripheral rings was measured across a cohort of 39 MS patients in order to compare the effectiveness of phase and QSM images in such identification. More detailed analysis was applied to a subset of six lesions with peripheral rings in order to establish the sources of the contrast in each image type, including comparison of the effects of SHARP and high-pass (SWI) phase filtering algorithms on phase and QSM contrast. Simulated field maps were generated for models of a solid and shell-like susceptibility distribution based on one lesion in order to illustrate the field patterns that such structures produce.

## Materials and methods

### Data acquisition

As part of a wider study of grey and white matter lesions, 39 subjects were scanned using a Phillips Achieva 7 T system equipped with a 16 or 32 channel receiver head coil. Of the 39 subjects, 19 were diagnosed with clinically isolated syndrome (CIS) suggestive of MS (at 4-year follow-up, 14 were diagnosed with clinically definite RRMS), eight were diagnosed with relapsing remitting MS (RRMS), nine were diagnosed with primary progressive MS, and three were diagnosed with secondary progressive MS. Data for susceptibility mapping were acquired using a multi-stack, spoiled, interleaved, 3D *T*_2_*-weighted FLASH sequence. Each image was acquired with four stacks, overlapping by 10 voxels at each interface, and 0.5 mm isotropic resolution (TE = 20 ms, TR = 150 ms, FOV = 196 × 164 × 85 mm^3^, EPI factor = 3, SENSE factor = 2, scan duration 8 min 49 s). The *T*_2_*-weighted magnitude and phase images from the four stacks were merged using software written in-house in MATLAB (Mathworks Inc., MA, USA). MPRAGE images were acquired for segmentation at 0.6 × 0.5 × 0.6 mm^3^ resolution and reconstructed at 0.5 mm isotropic resolution (TE = 5.89 ms, TR = 15 ms, TI = 1186 ms, shot interval = 3000 ms, FOV = 192 × 156 × 163.2 mm^3^, SENSE factor = 2).

### Phase processing

For all subjects, the phase images were first unwrapped using a Laplacian-based method [21]. Fields from sources located outside of the brain were removed using the SHARP method [18], with a spherical SHARP kernel with a radius of 3 voxels and a truncation value of 0.015. For the purposes of comparison, the phase data associated with the six lesions selected for detailed analysis were also unwrapped and filtered slice by slice in 2D using a standard high-pass filtering method employed in SWI [22]. Low-pass filtered images were first created by constructing a 2D square Hanning window in k-space, with filter widths of 0.094 (using the definition described by Walsh and Wilman [23]). The original data were complex divided by the low-pass filtered data to create unwrapped, high-pass filtered images. This filter width was chosen as a compromise between maximizing the removal of wraps and background fields in the data while preserving as much structural information as possible, based on the comparison of images from one data set using filter widths of 0.063, 0.094, 0.125, and 0.199.

### QSM reconstruction

A variety of different quantitative susceptibility mapping (QSM) techniques have recently been described based on different approaches to conditioning of the ill-posed phase-to-susceptibility inversion problem. These techniques can be divided into three main groups: (1) simple k-space division methods that make no assumptions about the form of the reconstructed QSM data [19, 24–26]; (2) iterative methods that use a priori information such as the edge information extracted from the associated magnitude data [27–30]; (3) methods involving rotation of the sample relative to the main magnetic field of the scanner, so as to allow acquisition of images of the sample at different orientations [17, 19, 28]. As there is no clear consensus about the nature of susceptibility offsets associated with MS lesions, the k-space division method was used here. Specifically, QSM data were generated from the masked, filtered phase data using the thresholded k-space division (TKD) method [25]. To condition the ill-posed nature of the QSM inversion due to zeros in the dipole kernel $\text{d}\left( \varvec{k} \right)$, his technique uses a modified Fourier domain dipole kernel ${\tilde{d}}\left( \varvec{k} \right)$, such that

$$\text{d}\left( \varvec{k} \right) = 3\cos^{2} \left( \beta \right) - 1 = 3\frac{{k_{z}^{2} }}{{k_{x}^{2} + k_{y}^{2} + k_{z}^{2} }} - 1,$$

where *β* is the angle between the *k*-space vector and *B*_0_, and

$${\tilde{d}}\left( \varvec{k} \right) = \frac{1}{3} \quad \text{when}\quad \left[ {\text{d}\left( \varvec{k} \right) \ge 0} \right]$$

$${\tilde{d}}\left( \varvec{k} \right) = - \frac{2}{3}\quad \text{when}\quad \left[ {\text{d}\left( \varvec{k} \right) < 0} \right] .$$

The filtered phase data are Fourier transformed, divided by ${\tilde{d}}\left( \varvec{k} \right)$, and then inverse Fourier transformed to yield a susceptibility map. Finally, the map is divided by a correction factor of 0.502 to compensate for the global underestimation inherent in this inversion method. This correction factor was chosen using the method described previously [25]. The field perturbation generated by a single voxel with a susceptibility of 1 ppm in a 7 T *B*_0_ field was generated using the forward calculation [31], and then this was inverted using TKD. The correction factor was then calculated by taking the ratio of the susceptibility of the voxel in the TKD-QSM to the “true” susceptibility of 1 ppm. This QSM method is computationally efficient, taking approximately 1 min to run on a PC (3.1 GHz Intel Core i3, 4 GB RAM, 64-bit Linux OS), including the unwrapping and filtering steps described above.

### Lesion selection

In order to establish the prevalence of peripheral rings in the cohort, a subset of up to 11 WM lesions were identified on the axial *T*_2_*-weighted magnitude images for each subject. Binary masks were generated using MRIcro (www.mricro.com), marking single, approximately central voxels in WM. In total, 305 lesions were identified in the 39 subjects. In addition, six well-isolated lesions with hyperintense peripheral rings in the axial phase images were identified across four subjects for more detailed analysis.

### Whole cohort lesion analysis

Following QSM processing, the lesions identified across all subjects were examined in the *T*_2_*-weighted magnitude, SHARP-filtered phase, and QSM images, in the axial, sagittal, and coronal planes intersecting the marked voxel. Lesions were not examined in the high-pass filtered phase images, as SHARP-filtered phase gives a more accurate representation of the fields generated within the ROI. The appearance of lesions in the phase and QSM images was classified as either (1) visible, showing contrast relative to normal appearing white matter (NAWM) with a hyperintense peripheral ring, (2) visible, showing contrast relative to NAWM without a peripheral ring, (3) showing no contrast, or (4) as unclassifiable. It was additionally noted whether there was a distinguishable external dipolar pattern surrounding the lesion.

### Testing clinical significance of ringed lesions

In order to establish any clinical significance of the presence of ringed lesions, 10 patients with at least one ringed lesion in the QSM images were selected for comparison with 10 patients with no such lesions. A Mann–Whitney *U* test was used to test for significant differences in age, disease duration, expanded disability status scale (EDSS), and multiple sclerosis severity score (MSSS) at the time of scanning.

### Detailed individual lesion analysis

In addition to the visual analysis described above, the six well-isolated individual lesions with hyperintense peripheral rings in the QSM images were further assessed in each image type by the generation of 1D profiles of mean voxel intensity in the lesion and surrounding white matter (WM). MPRAGE images were coregistered onto the *T*_2_*-weighted magnitude images using FLIRT in FSL. Lesion masks were drawn on the *T*_2_*-weighted images, and white matter masks were drawn on MPRAGE images using MRIcro. Profiles of the mean voxel intensity in the WM and in the lesion as a function of distance to the nearest point on the edge of the lesion mask were generated by convolving the voxels in the ROIs with a spherical kernel whose elements were set to the radial distance of the voxel from the centre of the kernel (rounded to the nearest integer). The distance of each voxel was set to the value of the smallest kernel element that overlapped with the lesion mask (for voxels outside of the lesion) or WM mask (for voxels inside of the lesion). The magnitude data were normalized to the mean voxel intensity 3 mm from the lesion edge. The QSM and phase images for each subject were normalized relative to the susceptibility and phase value in CSF averaged over two 6 × 6 × 10 voxel ROIs, one placed centrally in each ventricle. The MATLAB code used to generate these plots can be shared with interested labs on request.

One lesion with a hyperintense peripheral ring in the QSM image was used as a model to simulate the effect of a realistic susceptibility distribution on projected phase patterns. For that lesion, masks of the peripheral ring and of its central core were created from the *T*_2_*-weighted magnitude image data. These masks were assigned a nominal susceptibility of 0.15 ppm (the approximate susceptibility value of voxels in the ring in the QSM image of the lesion relative to the surrounding white matter) and the phase shifts caused by the resulting susceptibility distributions were modelled using the Fourier method [31]. The images were then processed with both high-pass and SHARP filtering methods to allow qualitative comparison with the measured phase shift patterns, and the SHARP filtered phase shift was inverted to form a susceptibility map using the TKD method.

## Results

### Optimization of Hanning window width for phase unwrapping and filtering

**Fig. 1:**
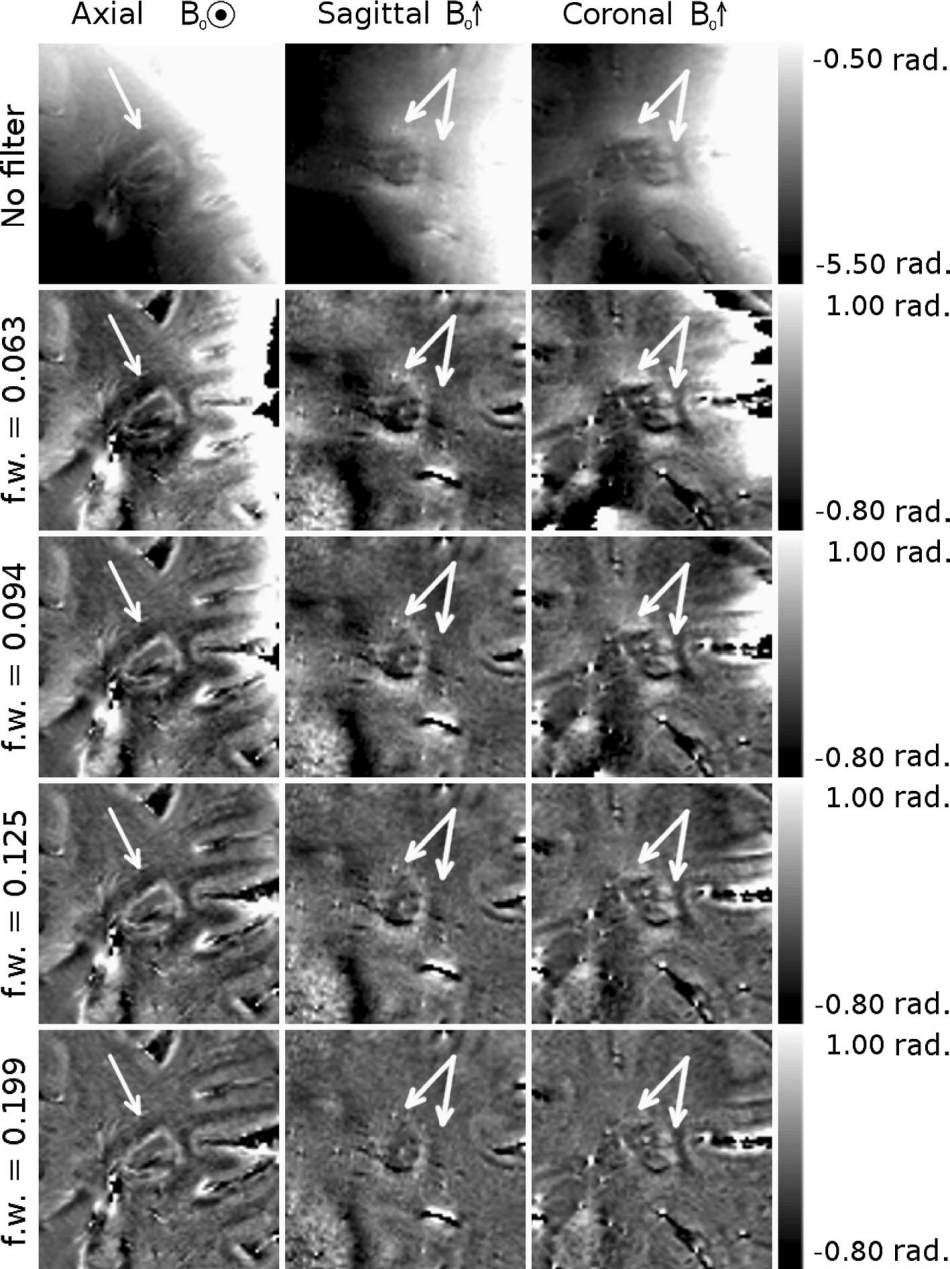
Phase images of a white matter MS lesion with a peripheral ring and visible external dipolar pattern (indicated by the *white arrows*). The raw, wrapped phase image is shown, along with images that have been filtered in *k*-space using Hanning windows with widths of 0.063, 0.094, 0.125, and 0.199

Figure 1 shows phase images of a WM lesion with visible external dipolar pattern. The phase was unwrapped using a Laplacian-based method with no filter applied in images shown in the top row, and unwrapped and filtered using a Hanning window with filter widths of 0.063, 0.094, 0.125, and 0.199 in images shown in the remaining rows. The external dipolar contrast is clearly present in the unfiltered image; however, most of the image is dominated by the large, slowly varying fields generated by field sources outside of the brain. As the filter width increases, the filtered images show remaining phase wraps visible on the right hand side of the axial and coronal images, as well as an overall flattening of the image and reduction in the visible external dipolar contrast as the range of spatial frequencies attenuated in the image increases.

### Detailed visual analysis of white matter lesions with peripheral rings

**Fig. 2:**
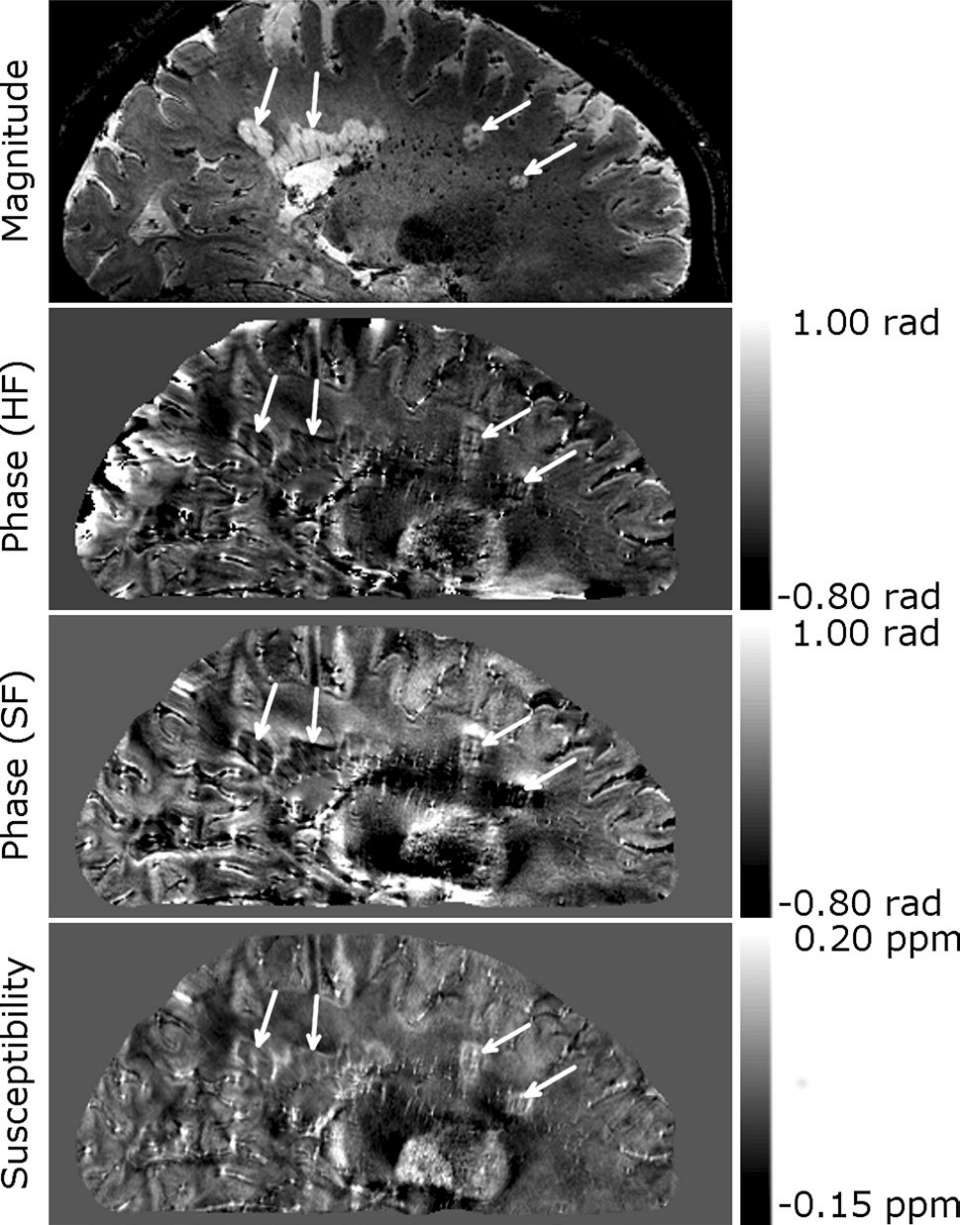
Whole head sagittal images of a patient with multiple sclerosis showing several white matter lesions, indicated by *white arrows*, with peripheral rings. Dipolar fields are apparent in the phase image, but do not appear in the QSM image. HF denotes images, which were unwrapped and filtered using a Hanning window. SF denotes images which were unwrapped with the Laplacian algorithm and filtered using the SHARP algorithm

Figure 2 shows whole-head sagittal images (magnitude, phase, and susceptibility contrast) from an MS patient. Several lesions of various shapes are indicated by white arrows. Dipolar projections can clearly be seen in the phase images. These projections are particularly apparent surrounding the two more spherical lesions on the right hand side of the image, where positive phase shifts are clearly seen above and below the lesions, and negative shifts are seen on either side. The contrast in the high-pass filtered (HF) phase is flatter than that seen in the SHARP filtered (SF) phase.

**Fig. 3:**
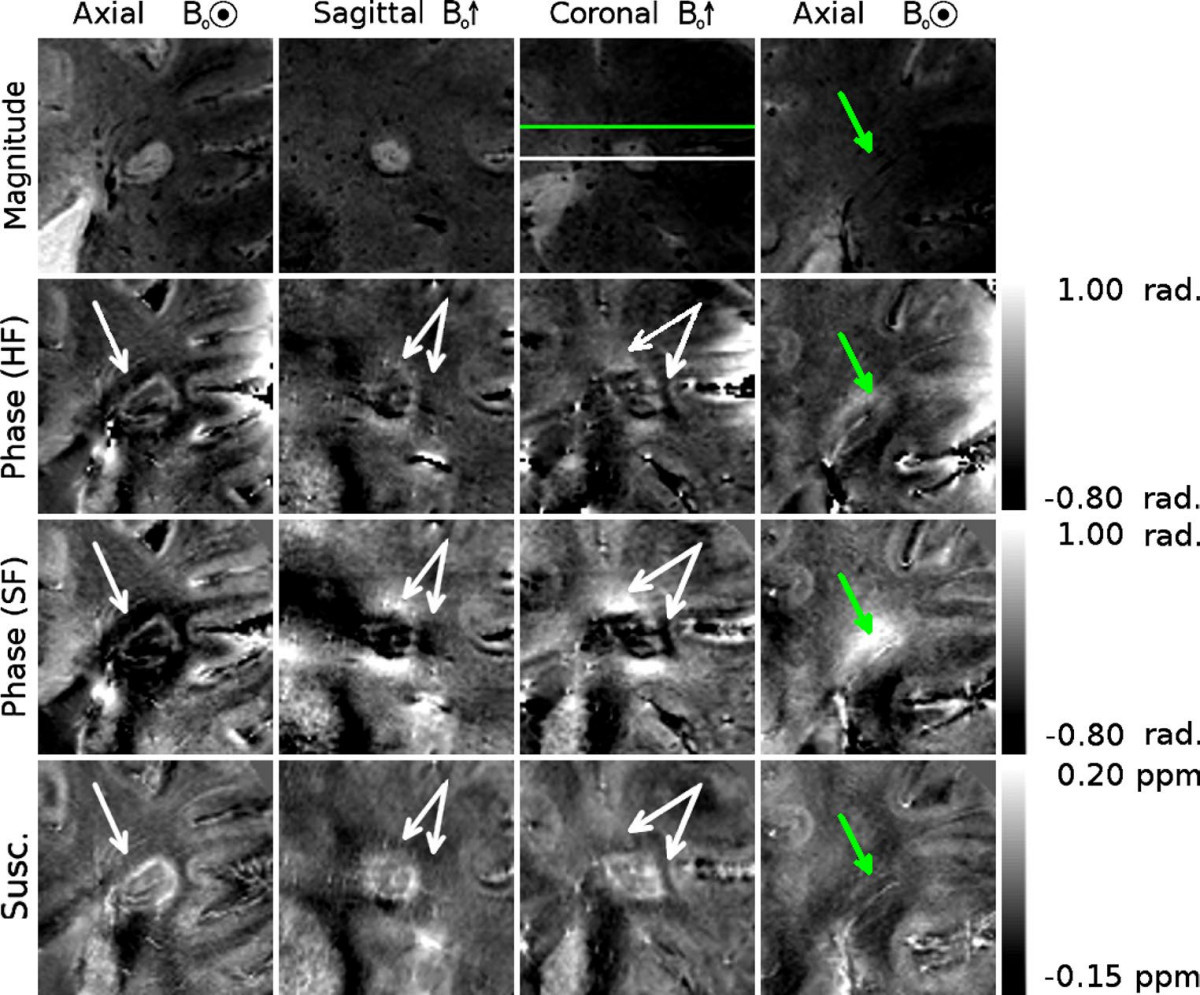
Magnitude, phase and QSM images of a white matter MS lesion with a peripheral ring. The first *three columns* show cross-sectional images through the lesions, with the plane of the axial images indicated on the coronal magnitude image in white, and with the location of the projected dipolar field lobes (identified from the phase images) indicated with *white arrows* on the phase and QSM images. A hyper-intense peripheral ring is visible on the QSM images. The *fourth column* shows axial images in the plane indicated in green on the coronal magnitude image, with the location of the projected dipolar field indicated with *green arrows* on the phase and QSM images

**Fig. 4:**
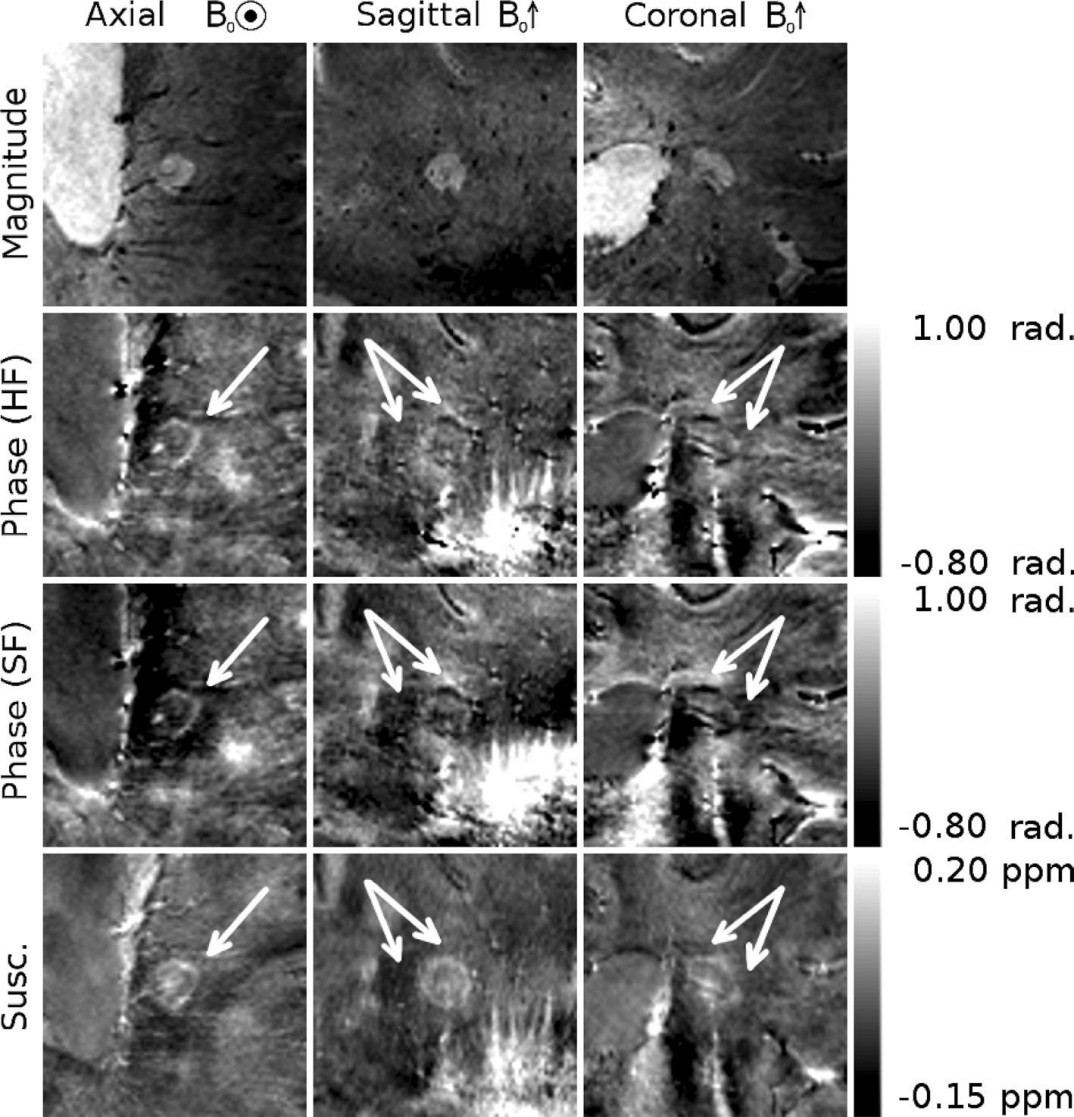
A second example of a white matter MS lesion with a peripheral ring. The location of the projected dipolar field lobes is indicated with *white arrows* on the phase and QSM images. A hyperintense ring can be seen at the periphery of the lesion in the QSM images

**Fig. 5:**
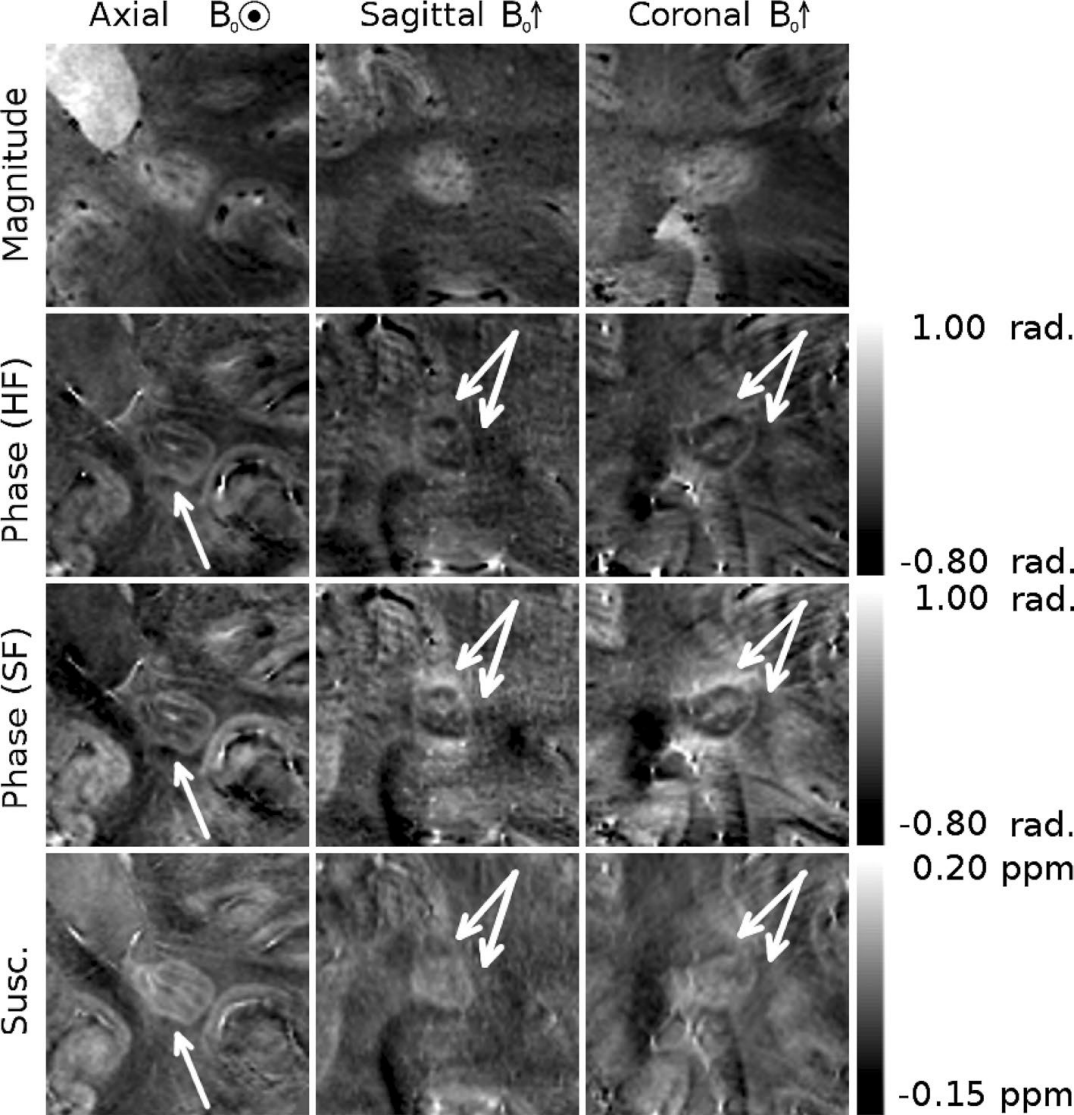
A white matter MS ring with a less distinct boundary in the magnitude image than those seen in Figs. 3 and 4. A weaker, but still present dipolar field is visible in the SF phase images, and its location is indicated by *white arrows* on the phase and QSM images. A hyperintense ring can be seen at the periphery of the lesion in the QSM images

Figures 3, 4, and 5 show different individual WM lesions in magnitude, SF and HF phase images, along with the corresponding susceptibility maps for axial, sagittal, and coronal slices cutting through the center of the lesion. In the axial plane, the SF images display hyperintense rings at the periphery of the lesion, consistent with the boundaries seen in the magnitude images. In Figs. 3 and 4, the ring in the axial SF image is surrounded by a distinct, hypointense region located outside of the lesion. In the sagittal and coronal images, the dipolar nature of the field perturbation underlying the phase contrast because the lesion is more apparent. Figure 3 also shows images of an additional axial slice located above the lesion. The magnitude image of this slice shows only normal-appearing white matter. In the SF and HF images, a hyperintense offset can be seen in the white matter region overlying the lesion. This hyperintensity corresponds to the region where the plane cuts the hyperintense lobe of the dipole field projected above the lesion. The susceptibility map shows no such offset. Figure 5 shows an example of a lesion with a less distinct boundary in the magnitude image. In the SF image, the dipole field is still present, and the hyperintense periphery is also evident in the susceptibility map. However, these features are less visually striking than in Figs. 3 and 4. Dipolar patterns in the phase are observed around all three lesions, extending significantly beyond the boundaries of the lesions observed in the magnitude images, as indicated by arrows. The intensity of these patterns is reduced in the HF images compared to the SF images due to the effect of the high-pass spatial filtering. The susceptibility maps show hyperintense rings at the lesion boundaries in all 3 planes, without significant external offsets. The presence of material with heterogeneous paramagnetic susceptibility is evident inside the lesions from the hyper-intensity seen in the susceptibility maps although this feature is slightly less prominent in Fig. 5. Corresponding hypointense regions within the lesions on the magnitude images indicates that this hyper-intense susceptibility contrast may be due to the presence of penetrating blood vessels in the lesions.

**Fig. 6:**
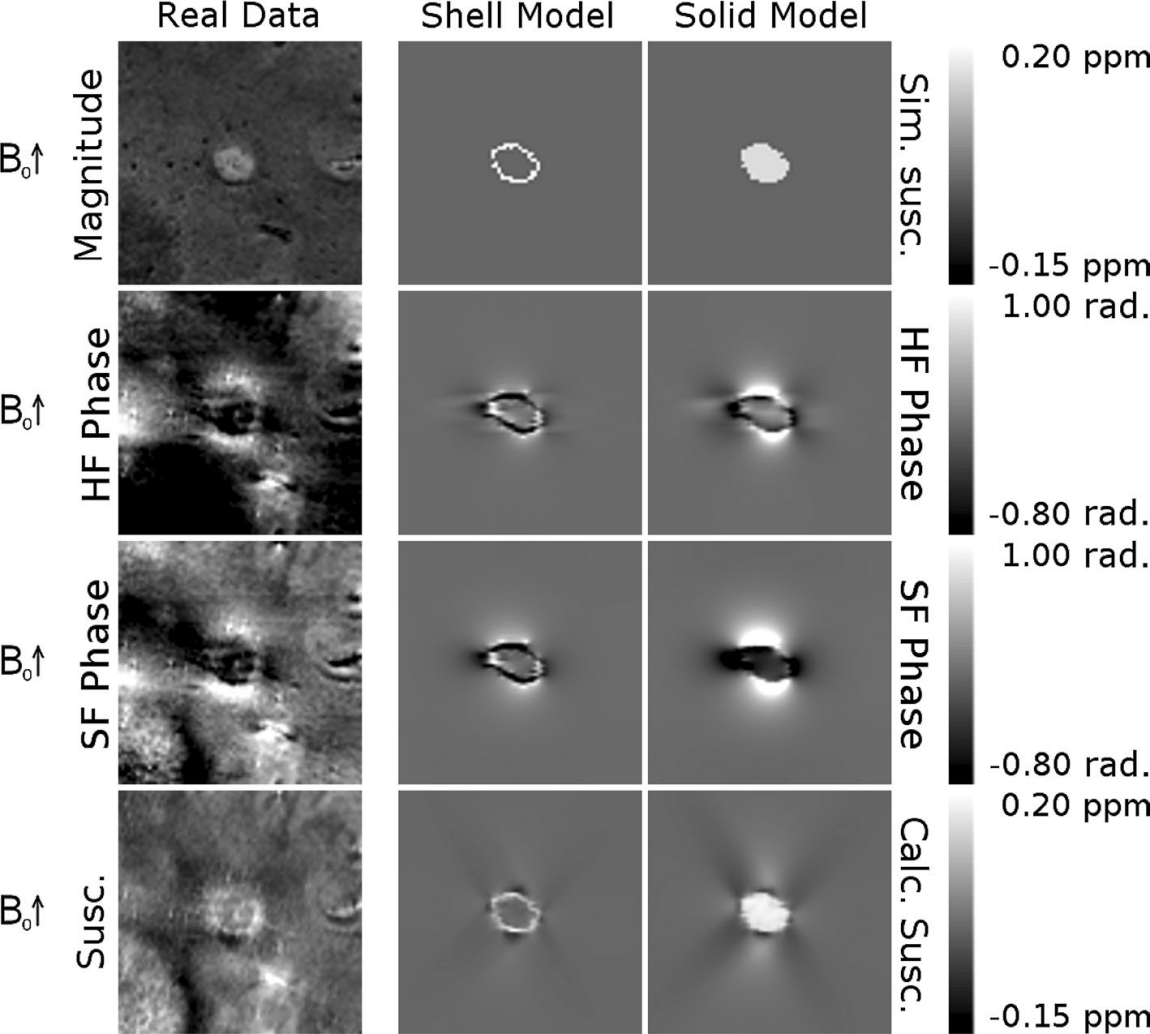
Sagittal susceptibility masks, simulated phase patterns, and QSM images generated using the TKD method, based on the lesion shown in Fig. 3. The *first column* shows corresponding experimental data. The *top row* shows susceptibility masks based on shell-like and solid representations of the lesion, shown in the *second and third columns*, respectively. The *second and third rows* show the result of a simulation of the high-pass filtered and SHARP-filtered phase data, respectively. The *fourth row* shows quantitative susceptibility maps generated from the SHARP-filtered phase data

Figure 6 shows simulated phase images produced from forward field calculations based on a mask of the peripheral ring of the lesion shown in Fig. 3 and on a solid mask of the entire lesion, in both cases assuming a constant susceptibility difference of 0.15 ppm in the mask region relative to the rest of the tissue. The simulated phase images were then processed with the same high-pass and SHARP filtering methods that were applied to the real data. Susceptibility maps were calculated from these phase images using the TKD method. Both the shell and solid models produced a dipolar phase pattern, shown in the sagittal plane, which is consistent with the pattern observed in the sagittal phase images in the measured data. The amplitude of the dipolar field is reduced in the HF phase compared to the SF phase, as observed in the real data. Additionally, some lateral distortion can be seen in the HF images. As would be expected, the external phase perturbation is larger in magnitude for the solid model since the dipole moment is stronger in this case. In addition, the solid model yields a more significant overall perturbation of the internal phase for this non-spherical lesion shape. In the shell model, while a dipolar perturbation is observed outside of the ring, there is also a phase shift within the shell itself of opposite polarity to the adjacent dipole lobe.

### Mean voxel intensity profile in ringed white matter lesions in magnitude, phase, and QSM images

**Fig. 7:**
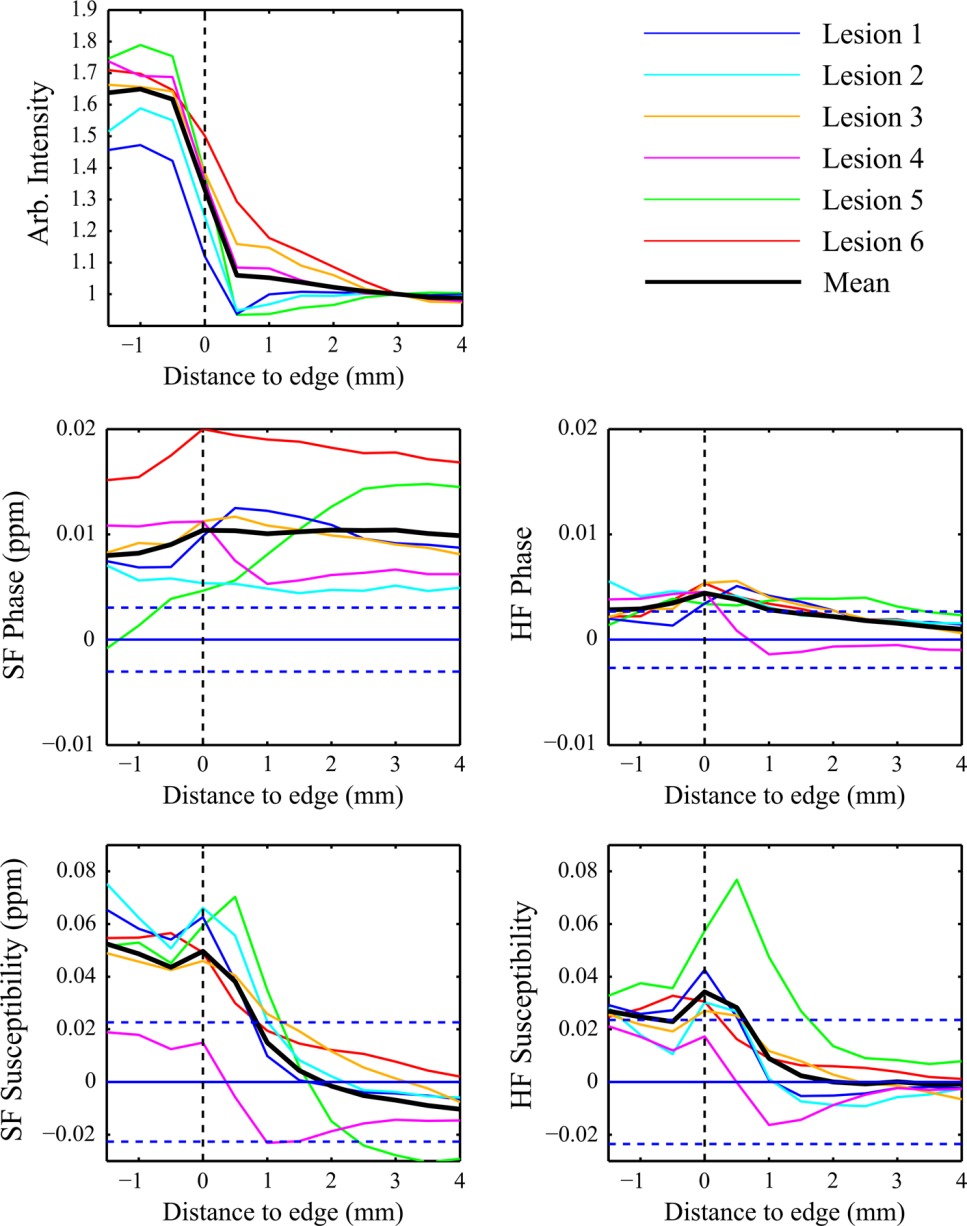
Profiles of the mean voxel intensity in the white matter as a function of distance to the nearest point at the edge of six lesions in: **a** *T*_2_*-weighted magnitude images, **b** SHARP-filtered phase images, **c** high-pass filtered phase images, **d** QSM images generated from SF phase data, **e** QSM images generated from HF phase data, showing individual lesion values (*coloured lines*) and mean values (*solid black lines*). Phase and QSM data were normalized relative to CSF, and the mean of the standard deviations calculated within the individual CSF ROIs is shown by the *horizontal dashed blue lines*

Figure 7 shows the mean voxel intensity as a function of distance from the lesion edge surrounding six individual lesions showing hyperintense contrast with peripheral rings, as well as the mean across the six lesions. Separate plots show profiles in the signal magnitude, high-pass (HP) and SHARP-filtered (SF) signal phase, and susceptibility derived from both HP and SF phase images. The mean and standard deviation in the CSF VOIs used to normalize the SF phase and SF phase-based susceptibility lesion profiles is shown in Table 1.

**Table 1:** Mean and standard deviation of phase and susceptibility in CSF ROIs

	Mean CSF SF phase ± SD	Mean CSF SF Susc. ± SD
Lesion 1 and lesion 2	−0.009 ± 0.003 ppm	−0.012 ± 0.017 ppm
Lesion 3 and lesion 4	−0.008 ± 0.003 ppm	0.001 ± 0.034 ppm
Lesion 5	−0.015 ± 0.004 ppm	0.003 ± 0.023 ppm
Lesion 6	−0.017 ± 0.006 ppm	−0.014 ± 0.034 ppm

In the phase and susceptibility plots, the mean across all patients of the standard deviation in the ROIs used to normalize the data is also shown by the dashed blue parallel lines above and below zero, giving an indication of the relative precision of measurements taken relative to these "zero" points. The magnitude profile is hyperintense inside the lesions, falling monotonically to a constant lower level in the external WM. The phase data show relatively flat mean profiles, with both the lesions and the external WM being hyperintense relative to CSF, and with consistent profiles for individual lesions. The mean SF phase profile is consistently higher relative to the CSF and has a greater variation between individual lesions than the HF phase. In contrast, the susceptibility profile has a consistent internal hyperintense offset, shows a peak at the lesion boundary, and falls monotonically to a constant level in the external WM.

The mean susceptibility profiles show a consistent trend, but the susceptibility variation seen in the data generated from the HF phase is lower in magnitude and flatter than the profile generated from the SF phase. The mean standard deviation across patients of the phase and susceptibility values in the CSF ROIs is of a similar magnitude for both the HF and SF data and is of significant amplitude relative to the mean profiles. This means that there is considerable uncertainty regarding the reference value used to normalize the data.

The individual lesion profiles show trends that are consistent with the mean profiles.

### Whole cohort lesion analysis

**Fig. 8:**
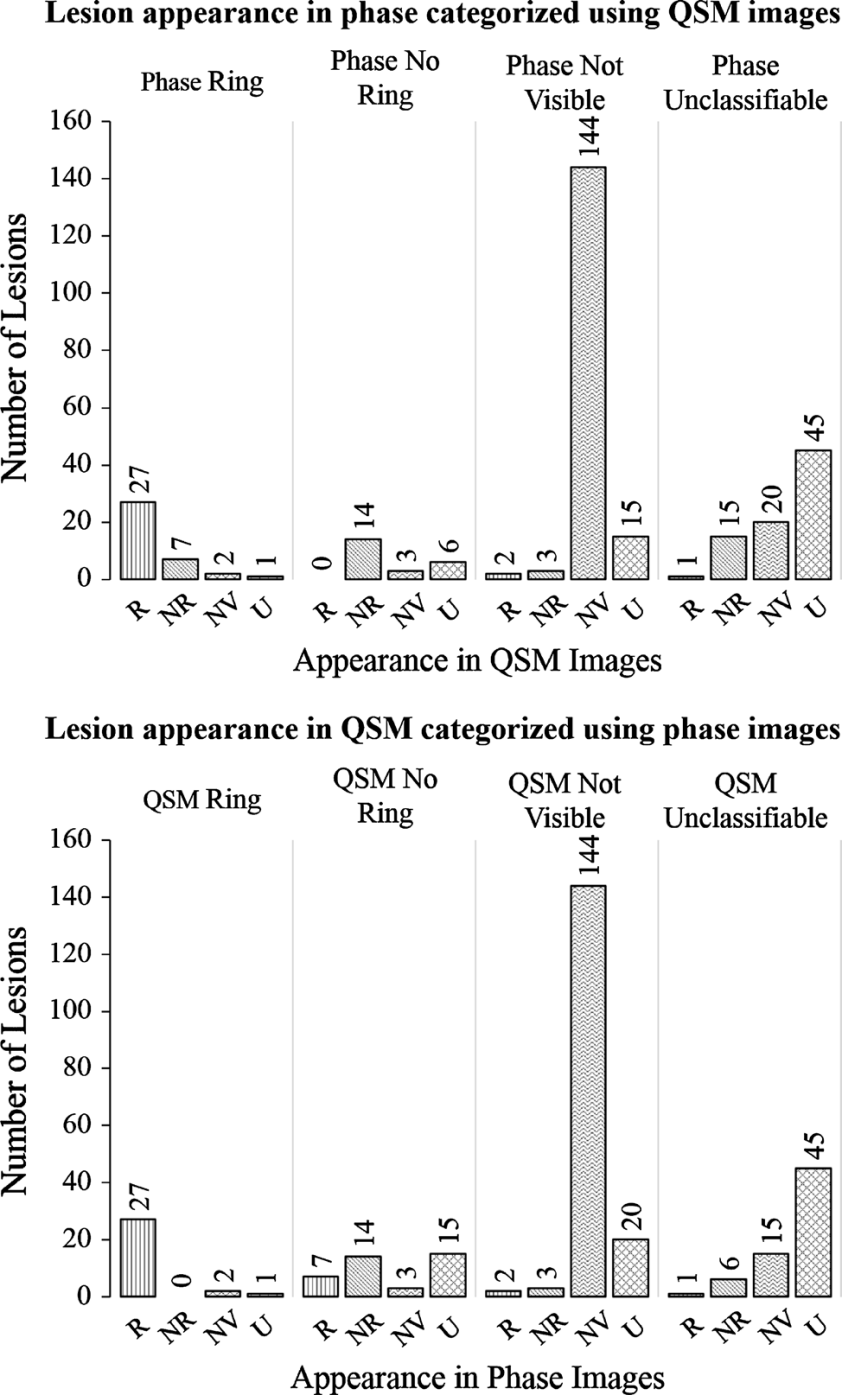
Appearance of lesions in SHARP-filtered phase images subdivided according to appearance in QSM, and appearance of lesions in QSM images sub-divided according to appearance in SHARP-filtered phase. Lesions were categorized as having a peripheral ring (R), having no peripheral ring (NR), not being visible (NV), or being unclassifiable (U) in SF phase and QSM images

Table 2 and Fig. 8 show the results of the analysis of the 305 lesions identified on the *T*_2_*-weighted magnitude images of the 39 subjects included in this study. Of these lesions, one was excluded as it was deemed unclassifiable in the sagittal and coronal planes of the *T*_2_*-weighted magnitude images. In all image types, lesions were considered unclassifiable if the contrast was not isointense, but the presence or absence of a focal lesion was ambiguous to the observer. Of the remaining 304 lesions, 60 (20 %) were visible in the SHARP filtered phase (SF) images and 69 (23 %) were visible in the QSM images. One hundred and forty-four lesions (47 %) were not visible on SF phase or QSM images. A further 81 (27 %) of selected lesions were unclassifiable in the SF phase images, and 67 (22 %) were unclassifiable in the QSM images. Of the 60 lesions visible in the SF phase images, 37 (62 %) displayed evidence of a peripheral ring, and 23 (38 %) had no peripheral ring. Nineteen (32 %) of the lesions visible in the SF phase images displayed visible, external dipolar contrast. Of the lesions visible in the QSM images, 30 (43 %) displayed evidence of a peripheral ring, 39 (57 %) had no peripheral ring, and no lesions displayed external dipolar contrast. Twelve (4 %) lesions were visible on SF phase images but invisible or unclassifiable in QSM data, 21 (7 %) were visible on QSM images, but invisible or unclassifiable on SF phase data. Forty-five lesions (15 %) were unclassifiable in both SF phase and QSM images. Of the 48 lesions visible in both SF phase and QSM images, 27 (56 %) had peripheral rings and 21 (44 %) had no rings on QSM, 34 (71 %) had peripheral rings, and 14 (29 %) had no rings on SF phase images. Of the 34 lesions with peripheral rings on SF phase images, 27 (79 %) had rings on the QSM images while seven (21 %) did not. Of the 27 of these 48 lesions in QSM images with peripheral rings, all had peripheral rings in the SF phase images.

**Table 2:** Appearance of lesions visible in SF phase and QSM

	Ring	No ring	Total
SF phase	37	23	60
QSM	30	39	69

**Fig. 9:**
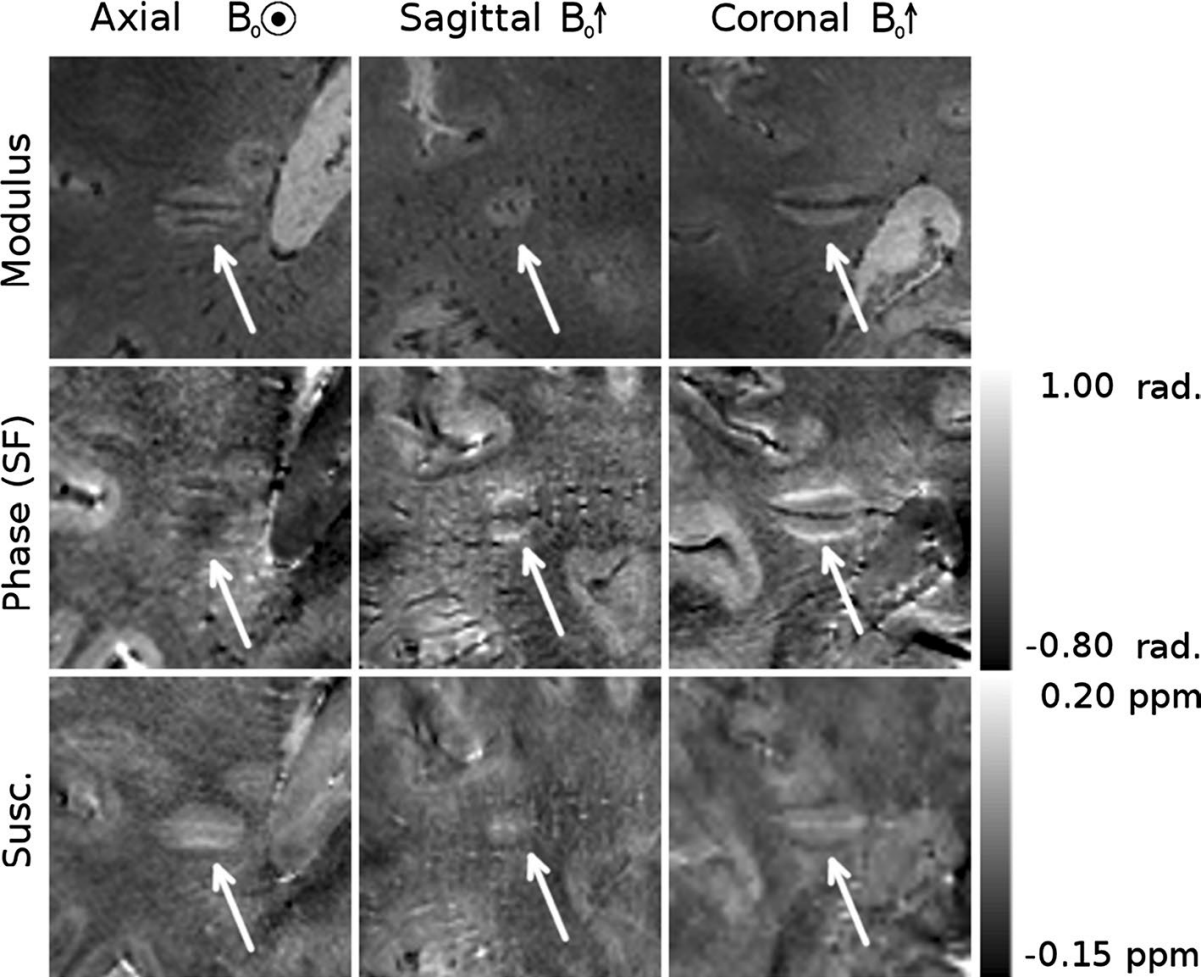
An example of a white matter MS lesion originally classified as having a ring in the SF phase image, but not in the QSM image. The lesion is indicated with a *white arrow* on the SF phase and QSM images. Positive phase offsets above and below the lesion give a ring-like appearance in the sagittal and coronal phase images; however, the QSM images reveal a relatively homogenous hyperintense contrast with no peripheral ring

As phase images were not expected to show anatomical features not present in QSM, the seven lesions, which were identified as having peripheral rings on the SF phase images, but not in the QSM images, were re-examined. Six of these lesions were homogenous and hyperintense in the QSM images (an example is shown in Fig. 9). The appearance of a peripheral ring in the phase was caused by positive dipole lobes at the lesion boundary, mostly apparent in the sagittal and coronal planes. One lesion was small, with a large central vein. The large, central vein obscured the peripheral ring in the susceptibility, while positive dipole lobes beyond the physical extent of the lesion accentuated the appearance of the ring in the SF phase.

### Clinical significance of ringed lesions

**Fig. 10:**
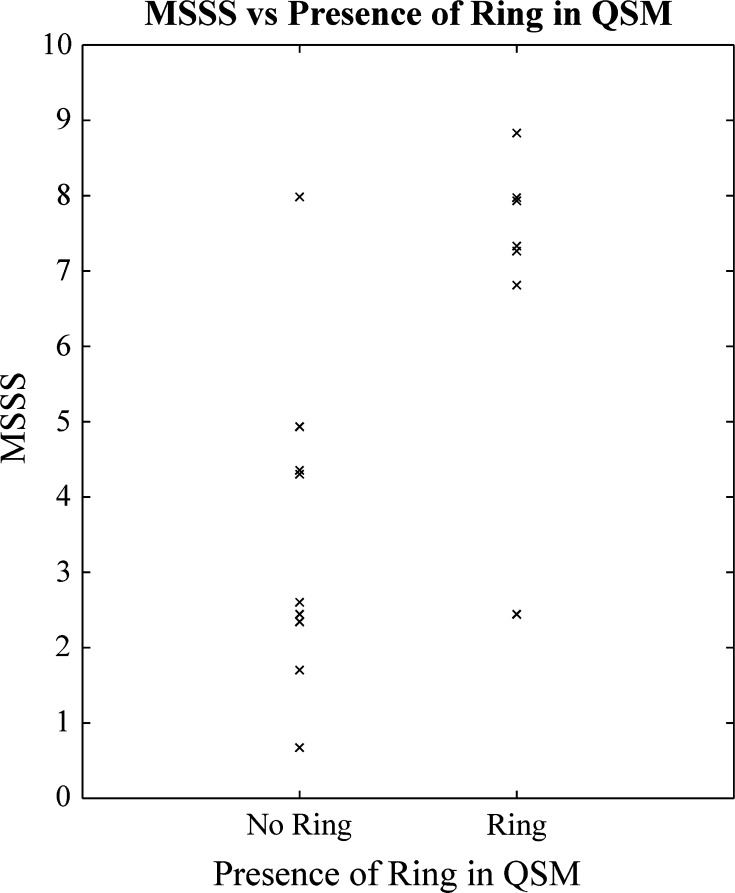
MSSS scores of the 10 patients with and 10 patients without ringed lesion(s) in the QSM images

Table 3 shows the mean, median, range, and *p* value derived from the Mann–Whitney *U* test of the age, disease duration (DD), EDSS, and MSSS at the time of scanning for 10 patients with and 10 patients without ringed lesions in the QSM images. No significant difference was found between patients with and without rings in age, DD, EDSS, or MSSS; however, there does appear to be a trend between the presence of rings and MSSS. The distribution of MSSS scores for the individual patients with and without rings are compared in Fig. 10.

**Table 3:** Differences in age; disease duration (DD); expanded disability status score (EDSS; and multiple sclerosis severity scale (MSSS) between 10 patients with and 10 without ringed lesions in QSM images

	Age (years) (mean/median/range)	DD (years) (mean/median/range)	EDSS (mean/median/range)	MSSS (mean/median/range)
Ring	42.55/39/32–62	5.33/5/0.4–25	3.55/3.5/1–6	6.62/7.30/2.44–8.83
No ring	37.10/37/20–59	9.41/3.4/0.8–31	2.7/2/0–6	3.93/3.45/0.67–7.98
*p* value	0.622	0.417	0.341	0.088

## Discussion

In this study, the use of ultra-high field MRI has allowed comparison of the appearance of white matter MS lesions with peripheral rings in high resolution magnitude, phase, and QSM images, as well as facilitating the analysis of contrast in the lesions using 1D plots of voxel intensity with respect to distance from the lesion boundary. Axial and coronal phase images showed obvious dipolar patterns surrounding some lesions: patterns that are not consistent with any expected distribution of iron in or around MS lesions if phase offsets are considered to be locally generated. Quantitative susceptibility maps showed lesion structures that are more consistent with the tissue changes known to occur in MS lesions from post-mortem studies [5, 32], where iron-bearing macrophages have been identified at the boundary of ringed lesions. Comparison between phase data that was unwrapped and filtered using a Hanning window and that which was unwrapped using a Laplacian-based method and filtered using the SHARP technique highlighted the sensitivity of both phase and QSM images to processing methods. Visual analysis of a subset of 304 lesions gave an indication of the prevalence of peripheral rings in phase and QSM images, and the variable morphology of individual lesions when their appearance is compared in phase and QSM images.

Histological studies have linked the paramagnetic rings surrounding some MS lesions to the presence of iron-rich macrophages at the periphery of the lesions [5, 10]. In vivo quantification of such features is clearly desirable, and the effect of such features on tissue susceptibility makes susceptibility-sensitive MRI contrast an obvious tool for such research. Using phase contrast rather than QSM to investigate changes in tissue susceptibility in MS poses the risk that images will be misinterpreted, especially if the lesion is not viewed in the sagittal or coronal planes where the non-local, dipolar nature of phase contrast is most obvious. For example, if phase contrast were simply used as a measure of the iron level surrounding these lesions, the presence of raised iron levels above and below the lesion would mistakenly be inferred, while reduced levels would be assigned to the region surrounding the lesion in central axial planes, as can be seen clearly in Figs. 3, 4, and 5. The exact appearance of lesions in the phase images depends on the spatial filtering that is applied to the phase data. The use of high-pass filtering reduces the amplitude of the phase variation due to the external field perturbations when compared to the SHARP filtered phase. However, as can be seen in Fig. 1, the degree of attenuation will depend on the size of the lesion relative to that of the filter window. Non-local effects generally also confound the use of the phase measured in a lesion relative to the phase of nearby normal appearing white matter as a means of characterizing tissue changes and mean that, in general, reliable quantitative information about tissue composition cannot be measured from local phase contrast.

In contrast, quantitative susceptibility maps show features that are consistent with the physical extent of the lesion and with the occurrence of normal appearing WM around the lesion. Locally elevated susceptibility can also be seen to occur with varying degrees inside the lesion and consistently in the peripheral ring, as expected from histology [5].

Iron levels cannot be directly quantified from a susceptibility map alone, particularly due to the negative contribution of myelin, which is diamagnetic, to the bulk susceptibility, although techniques for quantifying iron by combining information from susceptibility and R_2_* maps have been proposed [33, 34]. Recently, it has also been suggested that the presence of iron in tissue can be inferred from QSM images if the bulk susceptibility measured relative to that of the CSF are greater than zero [35, 36]. Both decreased myelin levels and increased iron levels lead to a positive increase in the net magnetic susceptibility [37–39]. Complete demyelination in a voxel would not be expected to increase the bulk susceptibility above that of CSF, and so any further increase in susceptibility is argued to be related to iron. As shown in Fig. 7, profiles through the lesions in *T*_2_*-weighted images show that *T*_2_* is increased inside the lesion and reduced at the lesion boundary before levelling off at the value for external normal appearing white matter. This variation is consistent with either myelin or iron loss within the lesion, but is most probably due to myelin loss and the destruction of normal tissue. On some *T*_2_* -weighted images (Figs. 3, 4, 5) hypointensity can be seen at the lesion edge, which would be consistent with increased iron (or myelin) in that region, although this is not detected on the averaged radial profiles, probably due to the competing effects of reduced myelination and increased iron deposition in this region. The average phase profile in both the HF and SF phase is slightly reduced inside the lesions, rising at the edge of the lesion, and remaining level (SF) or reducing slightly (HF) in the external WM. This gives little, if any, indication of a change in tissue composition internally or externally because of the non-local, dipolar nature of phase contrast [14]. In contrast, the susceptibility profiles are higher inside the lesion than outside, and display a peak at the lesion edge. When normalized to CSF, a positive susceptibility measured within the lesions is consistent with an internal loss of myelin and the presence of iron, and the peak at the lesion edge is suggestive of a peak in iron levels. The susceptibility profiles are therefore consistent with recently reported results from histology [5], which suggest that myelin levels are reduced inside lesions relative to the surrounding WM, while iron levels are increased at the lesion boundaries. The *T*_2_* weighted profiles are smaller in extent and are not obviously consistent with this description, but this may be due to the varying effects of iron and myelin in that signal

The magnitude of the phase variation relative to CSF was clearly reduced in the HF phase images when compared to the SF phase images. The SF phase in the lesions is consistently positive relative to the CSF; however, the mean standard deviation of the voxels in the CSF ROIs overlaps considerably with the phase profile in the HF data. This indicates both that normalized phase values are highly sensitive to the specific filtering applied to the data and that attempts to quantify phase variation relative to CSF are somewhat confounded by variability of values found within the "zero" region defined in the CSF.

The susceptibility calculated from the SF phase shows a more positive susceptibility within the lesion and a more negative susceptibility in the external WM than the susceptibility calculated form the HF phase. This may indicate a systematic underestimation of the magnetic susceptibility calculated using QSM due to the flattening effect of the high-pass filtering on the phase data. There is also a large variability in the susceptibility measured within the CSF, as shown by the large mean standard deviation in these regions. This indicates that even with the application of QSM, care must be taken in drawing conclusions from these measurements, as the “zero” point is poorly defined, so there is still a significant degree of uncertainty in the quantitative values measured.

Variation in susceptibility distribution can be seen between the lesions, for example, the smaller increase in susceptibility inside the lesion shown in Fig. 4 could be because of that lesion having undergone less demyelination than the lesions shown in Figs. 3 and 5.

Figure 6 shows the phase shift caused by a simulated shell of raised susceptibility at the periphery of a lesion and also that produced by a uniform increase in susceptibility of the region lying within the same lesion boundary. The simulated phase can be seen after both high-pass and SHARP filters have been applied. In both cases the resulting external phase shifts are consistent in polarity and orientation to the pattern observed in the real phase data. In the shell model, it can be seen that in addition to an external dipole, a local field shift is generated on the inner surface of the shell, with opposite polarity to the adjacent external dipole lobes. This feature can also be seen in the real phase images in Figs. 3, 4, and 5. The solid model produces a dipole of greater magnitude, but without this feature. The susceptibility of the shell and solid object were based on the susceptibility of the shells observed in experimentally acquired susceptibility maps, but the magnitude of the dipole produced by the shell model appears reduced compared to the real data, whereas the solid model generates a dipole of similar magnitude to the real data. As noted in the discussion of Figs. 3, 4, and 5, the amplitude of the dipolar phase patterns is reduced in the HF phase relative to the SF phase data. Additionally, some lateral distortion can be seen in the HF phase images as a result of the filter being applied in 2D to axial slices of the data, further demonstrating that images processed in this way must be interpreted with care. The calculation of susceptibility maps from phase images is an ill-posed problem, whereas the forward calculation used in the simulation is well conditioned, so this result strengthens the interpretations made from the QSM images and further illustrates need to study susceptibility maps rather than phase images when considering these lesions. The susceptibility distribution calculated from the simulated phase data is approximately consistent with the simulated susceptibility distribution, although there are some streaking and other artefacts present due to the imperfect inversion resulting from the truncated k-space filter used in the TKD implementation. This effect may account for the hypointense region seen immediately below the lesion in the susceptibility map, indicating that, even when using QSM, care must be taken when drawing inferences from small variations in contrast, despite the strong localization of contrast that QSM displays in comparison to phase imaging.

An indication of the relative prevalence and variable appearance of white matter lesions in SHARP filtered phase and QSM images can be seen in Table 2 and Fig. 8. Of the 304 lesions marked on the *T*_2_*-weighted magnitude images, 20 % were visible in phase images. This is lower than the prevalence reported in previous studies which included similar analysis [7, 10, 32, 40], in which 40–78 % of lesions in magnitude data were found to be visible in phase images. These figures could be affected by a number of factors, including the field strength of the scanner used, the resolution of the data acquired, the criteria by which lesions were identified for comparison and the number of planes in which lesions were compared. Further variation would be expected due to the subjective nature of visual comparison and the lack of consistent criteria for categorization of lesions across different studies. Our data were acquired at 7 T with a high isotropic resolution of 0.5 × 0.5 × 0.5 mm^3^, and datasets were compared in the axial, sagittal, and coronal planes. In contrast, images used for lesion identification in previous studies were typically acquired with high in-plane resolution, but with slice thicknesses of 2–3 mm [7, 10, 32, 40], and only axial images were reported. In one case the lesions used for comparison were specifically selected for their large size [40]. For this reason, smaller lesions (<3 mm in diameter) would be less likely to be selected for comparison. A systematic bias towards larger lesions in previous analyses may explain the increase in the proportion of lesions found to be visible in phase images, as smaller lesions may have different levels or distributions of iron deposition or demyelination. As small lesions may be early indicators of new disease activity, their investigation has been recognized as an important area of future focus [40], making the use of high, isotropic resolution acquisitions an important improvement on previous imaging protocols.

The absolute number of lesions identified was greater in the QSM images than in the phase data. The majority of this difference is due to lesions with no ring in the QSM images, and lesions that are unclassifiable in the phase images. In contrast, the majority of lesions which displayed rings in the QSM data also appeared in the phase images. This suggests that lesions with peripheral rings are more likely to have a relatively consistent appearance in the phase and QSM images, possibly indicating that the changes in tissue composition in these lesions is greater relative to the surrounding white matter than lesions with no ring. Lesions with less pronounced changes in microstructure relative to the surrounding white matter may be more obscured by the non-local dipolar projections inherent in phase imaging, making them unclassifiable or undefined, while the correction of these projections in QSM may allow a greater proportion of such lesions to be identified.

Of the 48 lesions visible on both phase and QSM images, 27 (56 %) had rings on both phase and QSM images, 14 (29 %) did not display rings in the phase or QSM images, seven (15 %) displayed rings in the phase, but not the QSM images, and no lesions displayed rings in the QSM images, but not in phase images. Recently published work has highlighted that solid, nodular distributions of magnetic susceptibility can lead to shell-like patterns in phase images [40]. Such an effect appears to explain most of the lesions found in our data which were categorized as displaying rings in the phase images, but not in QSM images, with an example shown in Fig. 9. In contrast, the fact that no ringed lesions were found in QSM images where no ring had been found in the phase data suggests that QSM may offer improved specificity in the identification of peripheral rings in white matter lesions compared with phase images. This further highlights the importance of using QSM when trying to quantify such features.

Since our data were acquired during a single visit for each patient and without the use of contrast, our ability to test any significance of the presence or absence of peripheral rings on the severity of progression of MS symptoms was limited. As discussed above there was a trend for the presence of rings to be associated with MSSS score, but the sample size used for this comparison was relatively small, and so while no link between peripheral rings and MSSS can be inferred from the results presented here, such a link is worthy of future study.

## Conclusions

In this study, isotropic high-resolution, whole head *T*_2_*-weighted images acquired at ultrahigh field were used to compare phase imaging and QSM as a means of investigating white matter MS lesions with peripheral rings. Phase images were shown to be dominated by non-local dipolar field effects, causing both positive and negative shifts in the contrast, even in the axial plane. These effects have the potential to be misleading when interpreting phase images and preclude the use of phase contrast in studying tissue composition. However, QSM techniques can be applied to phase data to yield susceptibility maps showing contrast that is much more closely linked to the local tissue composition. Although the susceptibility values are affected by both myelin and iron, the use of susceptibility maps in combination with *T*_2_*-weighted data allow inferences to be drawn about changes in tissue composition and comparison to be made to histology. The peripheral rings and visible dipolar field pattern apparent in some phase images were shown to appear in both SHARP and high-pass filtered data, although high-pass filtering was found to systematically yield lower susceptibility values both inside and outside of lesions in the resulting QSM. The prevalence of peripheral rings in phase and QSM data was found to be lower than previously reported [7, 10, 32, 40]; however, this could be attributed to the high isotropic resolution images used in this study, which may have resulted in the identification of smaller lesions in the magnitude data than those identified in other studies.
